# Dual paths cryptosystem based on tilt Fresnel diffraction using non-spherical mirror and phase modulation in expanded fractional Fourier transform domain

**DOI:** 10.1038/s41598-019-50263-4

**Published:** 2019-10-21

**Authors:** Hang Chen, Zhengjun Liu, Camel Tanougast, Feifei Liu, Walter Blondel

**Affiliations:** 1 School of Electrical Engineering and Automation, Jiangxi University of Science and Technology, Ganzhou, 341000 China; 2 Centre de Recherche en Automatique de Nancy (CRAN-CNRS, UMR 7039), University de Lorraine, Nancy, 54000 France; 3 Department of Automation Measurement and Control, Harbin Institute of Technology, Harbin, 150001 China; 4 School of Electrical Engineering and Automation, Jiangxi University of Science and Technology, Ganzhou, 341000 China

**Keywords:** Optics and photonics, Physics

## Abstract

In this paper, a dual optics paths optical image cryptosystem based on tilt Fresnel diffraction and a phase modulation in extend fractional Fourier transform (eFrFT) domain is presented. The tilt Fresnel is designed by using a non-spherical mirror. A part of data from the original image is modulated by the mirror, while the other part is encoded by an expanded fractional Fourier transform. Besides, the random data of the dual channels is combined for forming the encrypted image. The structure parameters in designing the optical hardware system and the random phase can be regarded as decryption keys. Various potential attack experiments are implemented to check the validity of the proposed cryptographic system.

## Introduction

With the rapidly development of multimedia application, the issue of security in transmission and storage of the confidential image has becoming more and more importance. Optical technologies have become increasingly attractive and extensively developed on information securing since the double random phase encoding (DRPE) was first reported by Refregier and Javidi first proposed in 1995^1–13^. One of the motivations is that the characteristics of fast computing and parallelism of optics are significant in real-time applications. Besides, the various complex degrees of freedom offered by optics makes it possible to encode/decode data in secure way. In recent years, many optical image cryptosystem based on different optics information means, such as optical transform^2^, holography^3^, diffraction^4,5^, polarization^6^ and ptychography^7^ have been reported. In order to deal with the problem caused by distribution and management of secret keys, some asymmetric optical information cryptosystems have been proposed by using phase truncation^9,10^, Yang-Gu algorithm^11^ and equal modulus decomposition^12,13^. In addition, an information authentication system has been reported by using interference between two beams in gyrator domains^14^. In the ref. ^14^, the performance of the system in resisting some potential attacks has verified the robustness of the proposed algorithm. Recently, combing Fresnel diffraction and a phase modulation in FrFT domain, an novel optical encryption system for color image is presented^15^. However, in most of the optical cryptosystems mentioned above, including symmetric and asymmetric scheme, the beams are modulated by a single optics information mean in one beam path, one optical transform, for instance. To our best knowledge, the cryptosystem based on dual optics paths has not been reported yet.

In this paper, combing tilt Fresnel diffraction and a phase modulation in eFrFT domain, we present a security-enhanced encryption technique for optical image. The beam is split by a beam splitter into two optics paths after the secret image is encoded by spatial light modulator (SLM). A part of data from the secret image is encoded by an expanded fractional Fourier transform. The other part of the image is modulated by the non-spherical. Finally, two beam paths convergence at the output plane of the cryptosystem. The random data of the two channels is combined for forming the encrypted image. To verify the validity and capability of the proposed encryption technique, a series of experimental results are given in the following step.

## The Cryptographic System

Our scheme begins with the concept of double optics paths encryption by using a beam splitter. In first path, the secret image is encoded in extended fractional Fourier transform^16^. The other path, the optical process can be expressed by employing phase modulation and tilt Fresnel diffraction alternately. With the help of the interference by a wave generated from one random mask, the secret signal propagated across the extended fractional Fourier transform lens is recorded and encrypted simultaneously. To simplify the expression, we use one-dimensional representation in the following explanation. Here, we suppose the random phase function is *φ*(*x*) and it distributed uniformly in the interval [0,2*π*]. Referring to^16,17^, the extended fractional Fourier transform can be expressed as follows:

1 $$\begin{array}{rcl}F(u) & = & K\int f(x)\exp [i\varphi (x)]\\  &  & \times \exp [i\pi \frac{({a}^{2}{x}^{2}+{{\rm{b}}}^{2}{u}^{2})\cos \,\phi -2ab(xu)}{\sin \,\phi }]{\rm{d}}x\end{array}$$

where *f*(*x*) and *F*(*u*) represent the input and output function of the transform, respectively. The symbols *a*, *b* and *φ* are three parameters of extended fractional Fourier transform, while K is a complex constant. These parameters can be expressed by the physical parameter of the optical setup: wave length *λ*, focal length *f* of the lens and the propagate length before and after the lens *l*_1_ and *l*_2_. The mathematical definition can be described as follows:

2 $${a}^{2}=\frac{1}{\lambda }\frac{\sqrt{f-{l}_{2}}}{\sqrt{f-{l}_{1}}}\frac{1}{\sqrt{{f}^{2}-(f-{l}_{1})(f-{l}_{2})}}$$

3 $${b}^{2}=\frac{1}{\lambda }\frac{\sqrt{f-{l}_{1}}}{\sqrt{f-{l}_{2}}}\frac{1}{\sqrt{{f}^{2}-(f-{l}_{1})(f-{l}_{2})}}$$

4 $$\varphi =\arccos (\frac{\sqrt{f-{l}_{1}}\sqrt{f-{l}_{2}}}{f})$$

**Figure 1:**
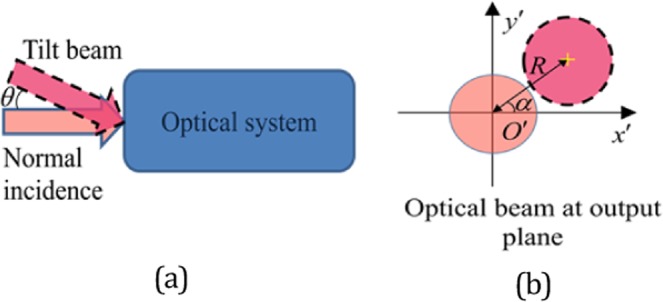
The model of tilt diffraction: (**a**) The tilt beam and normal incidence and (**b**) The spot shift at the output plane.

For the other beam path, the half secret image is irradiated by a uniform beam and propagated with distance d1 into a random phase mask ${\phi }_{2}(x,y)$. Thus, the left side light filed of the mask can be expressed as

5 $$L1=\exp [i\phi (x,y)]{F}_{d1.\lambda }[I(x,y)]$$

where *F* denotes the operation of Fresnel diffraction and the parameter *λ* is the wavelength as mentioned above. Thereafter, with the help of a non-spherical mirror, the beam continues propagate with distance d2 and the finally tilted achive the CCD.

6 $$\begin{array}{rcl}L2 & = & {F}_{d2.\lambda }(L1.\ast dp)\\ dp & = & \exp (i\frac{2\pi }{\lambda }\cdot [n0-1]\cdot z\cdot )\\ z & = & [x\cdot \,\cos (\alpha )+y\cdot \,\sin (\alpha )]\cdot \,\tan (\theta )\end{array}$$

where *dp* represents the phase delay of tilt Fresnel diffraction. The symbols *α* and *θ* are the angle parameter in tilt diffraction as shown in Fig. 1.

**Figure 2:**
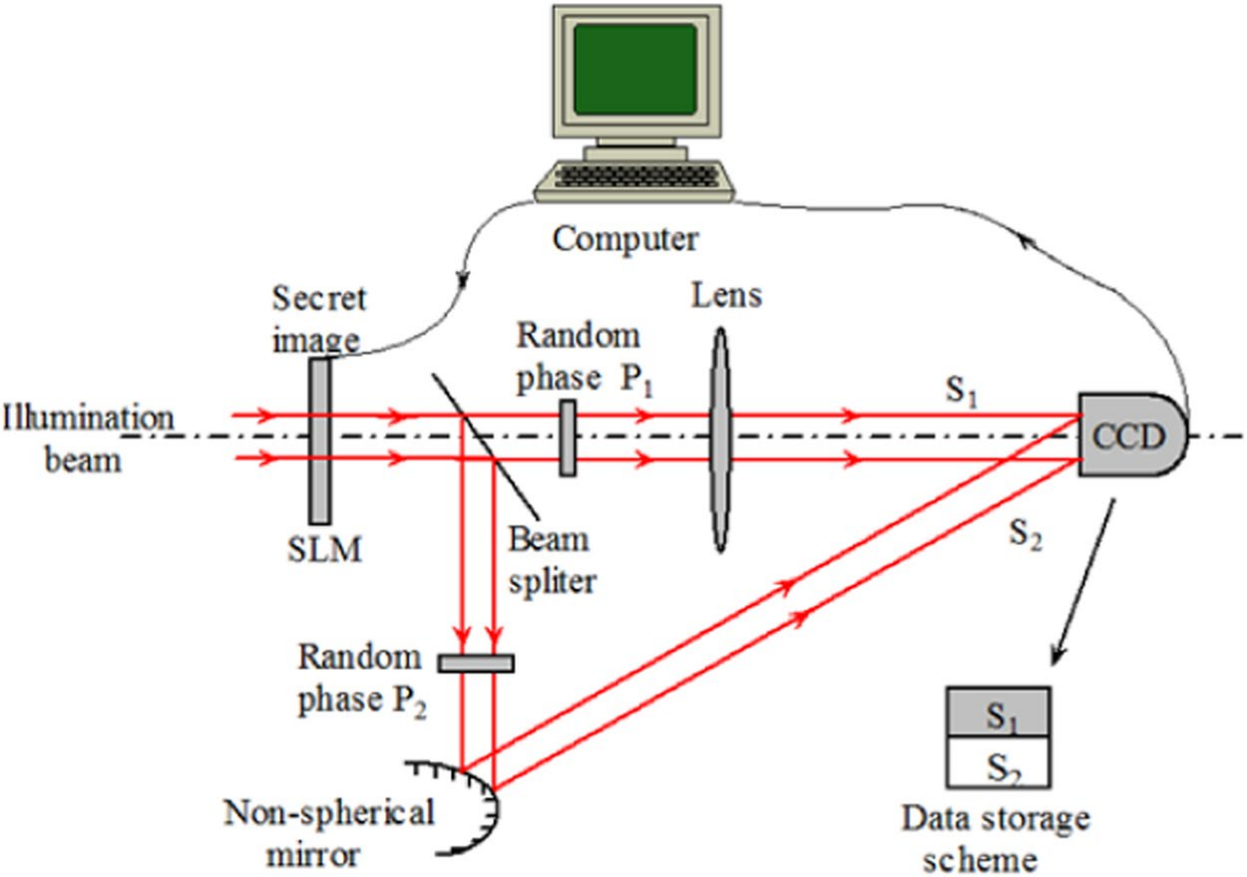
The electro-optical setup of the proposed cryptosystem.

The corresponding electro-optical setup for the proposed cryptosystem is depicted in Fig. 2. By using the spatial light modulator (SLM) controlled by a PC, the secret image is modulated by a uniform beam. As we can see from Fig. 2, the beam propagates into two paths *S*_1_ and *S*_2_ and finally the beams reaches the output plane and recorded by CCD. The secret data is encoded by two optical paths and storage in different part of the plane, respectively. In the output plane of the cryptosystem, the off-line holography technique is implemented for recording the phase data. In the decryption approaches, the encrypted image can be retrieved by the inverse optical system composed by inverse extended fractional Fourier transform and inverse tilt Fresnel diffraction, while the phase mask is set in the light path. For the correct decryption, the conjugate phase make placed in the light path need to be exchanged due to the reverse calculation.

**Figure 3:**
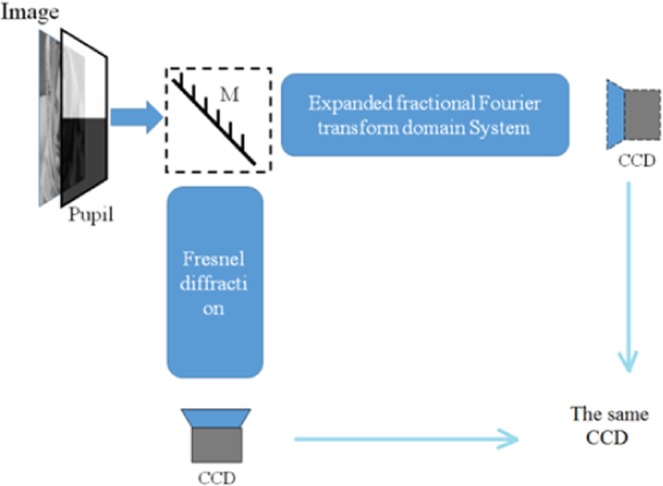
The control of sub-images for encrypting.

To avoid the cross-talk effect, the eFrFT and Fresnel diffraction are divided into different optical structures. As shown in Fig. 3, two sub-images are selected with a pupil by turning on and off to enter two encryption optical units. This sub-image hiding scheme is to make a multifarious optical encryption, since some encryption approaches were designed by single transform or several transforms in serial state.

## Numerical Simulations

**Figure 4:**
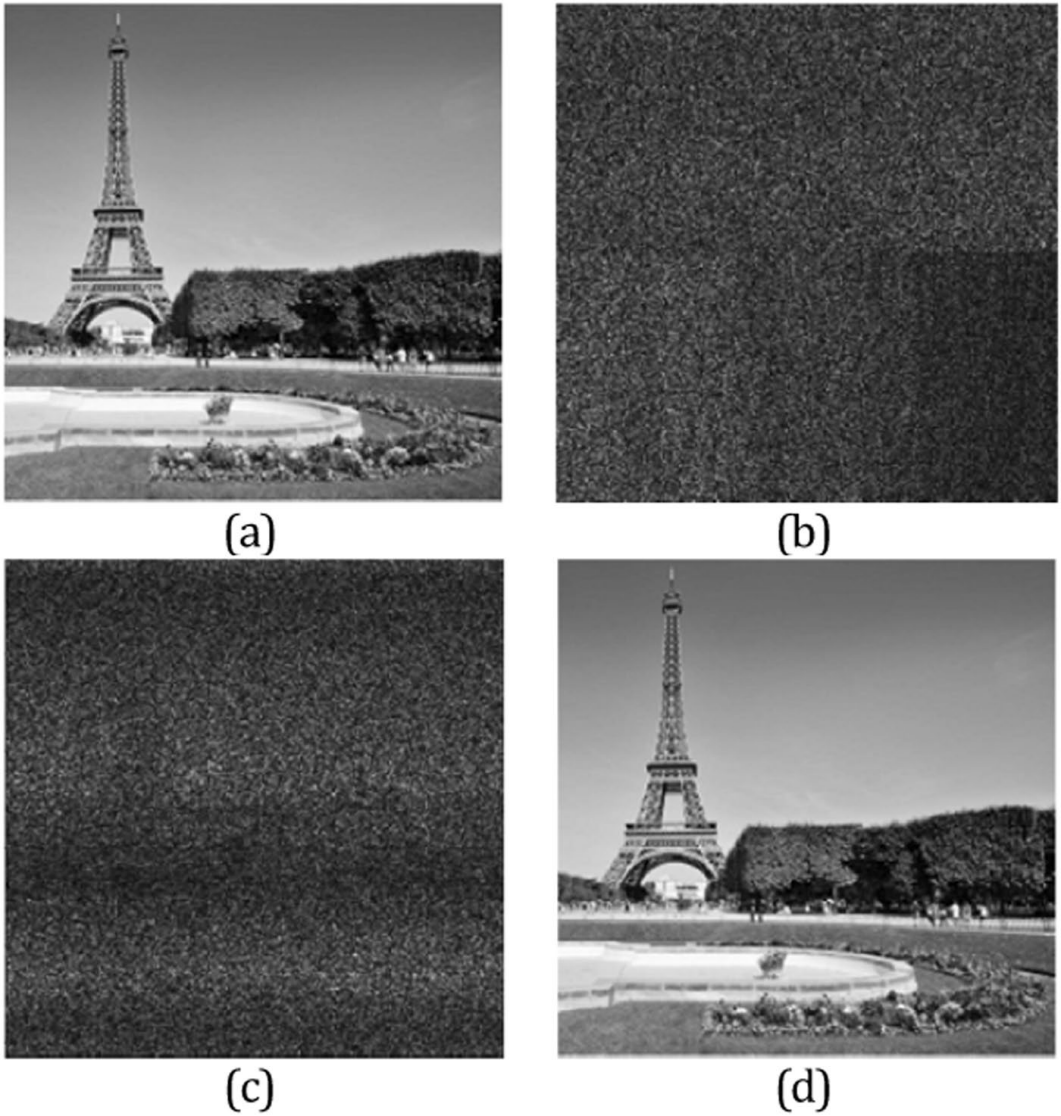
(**a**) Original color image; (**b**) encrypted image; (**c**) fail decryption with fake keys; (**d**) decrypted image with correct keys.

In this section, the numerical simulations are given to verify the validity and security of the proposed cryptosystem. One grayscale image taken from Paris Eiffel Tower, which has a size of 256 × 256 pixels, is chosen to as the secret image. In the experiment, the wavelength of the beam is fixed in 632.3 nm. Besides, the focal length of the lens is set as 12 cm. By employing the parameters mentioned above, the secret image is encrypted by the proposed cryptosystem effectively and the image before and after encryption are illustrated in Fig. 4(a,b), respectively. Figure 4(c) shows the fail decrypted result using the fake keys. In calculation, the top part and the bottom part decryption approaches are performed by using fake propagation length and propagation wavelength, respectively. As shown in Fig. 4(c), the decryption result is almost a noise image and the secret information cannot be recognized by human eyes. By using the inverse optical system, the original image can be retrieved with all the correct parameters and the retrieved pattern is depicted in Fig. 4(d).

For the robustness and security analysis, some numerical simulation of occlusion attack^18^ and known-plaintext attack^19^ are performed. Before the security analysis, the peak-signal-to-noise ratio (PSNR) is presented firstly to estimate the similarity between the plaintext and ciphertext of the proposed cryptosystem. The equation interpretation of PSNR can be written as follows

7 $$PSNR({I}_{{\rm{d}}},{I}_{0})=10{\log }_{10}\frac{{255}^{2}M\times N}{\sum _{\forall x,y}{[{I}_{{\rm{d}}}(x,y)-{I}_{0}(x,y)]}^{2}}(\mathrm{dB}){\rm{.}}$$

where the symbol *I*_0_ and *I*_*d*_ denote the secret image and decrypted data. The parameters *M* and *N* are the sizes of the function. In this experiment, the PSNR value between the original image and encrypted (correct decrypted) image are 5.9375 and 253.9735. Note that the value of PSNR large than 50 indicates that the difference between the two images is unrecognizable.

**Figure 5:**
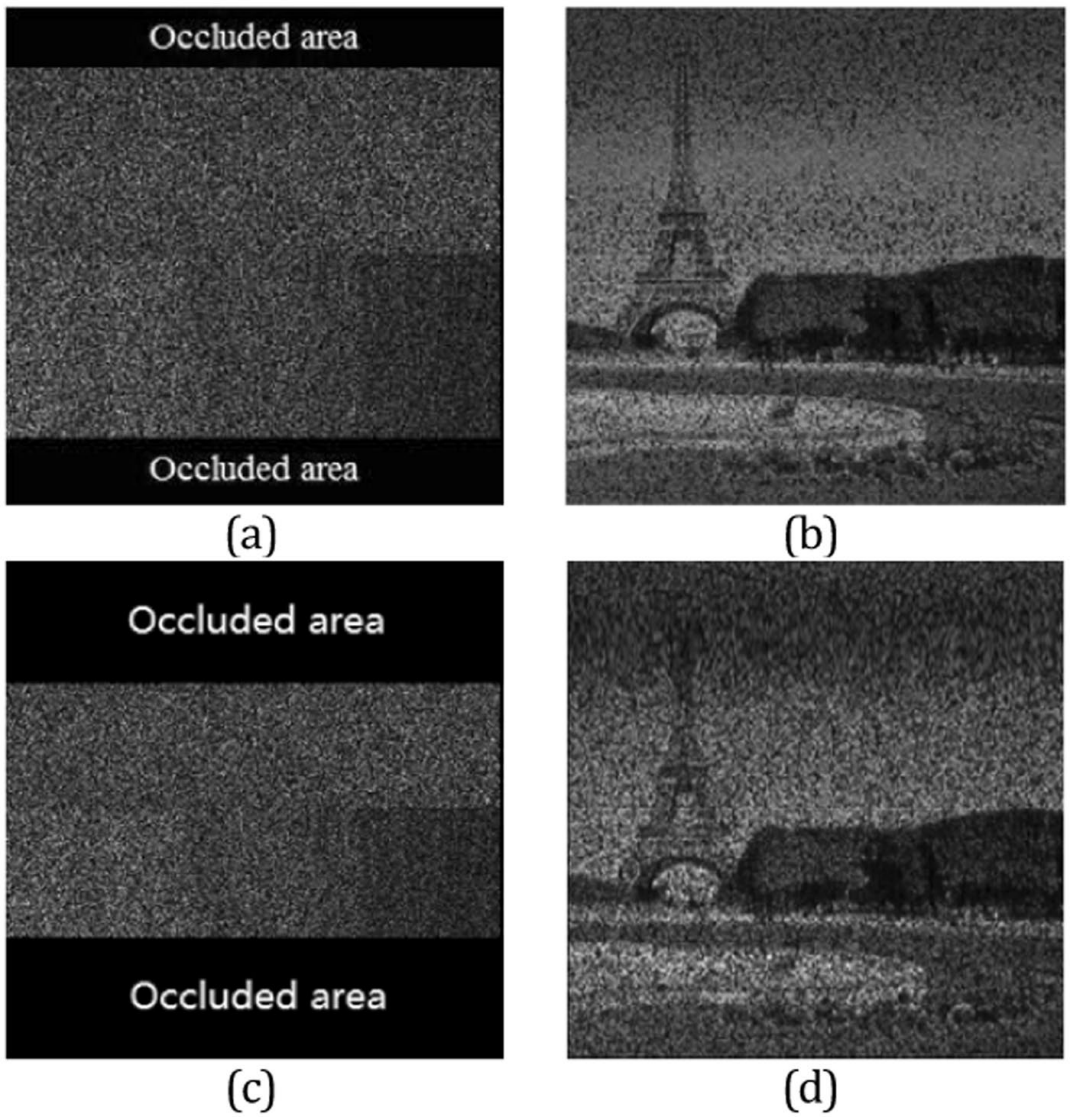
The test of occlusion attack: (**a**) the occluded encrypted image; (**b**) the recovered image; (**c**) the second occluded encrypted image and (**d**) the retrieved image of (**c**).

The correct decryption approach is employed with the partly occluded original image, which is depicted in Fig. 5(a). Here we emphasize that the occluded pixels of the data are filled with the number 0 in simulation and the corresponding result is displayed in Fig. 5(b). A worse situation is considered as larger part occluded attack, the occluded image and the corresponding attack result are given in Fig. 5(c,b), respectively. As shown in Fig. 5(c,d), the outline information of the original input image can be recognized in vision. Apparently, the decrypted data depicted in Fig. 5(b) has higher quality than the other one due to the smaller occluded area.

**Figure 6:**
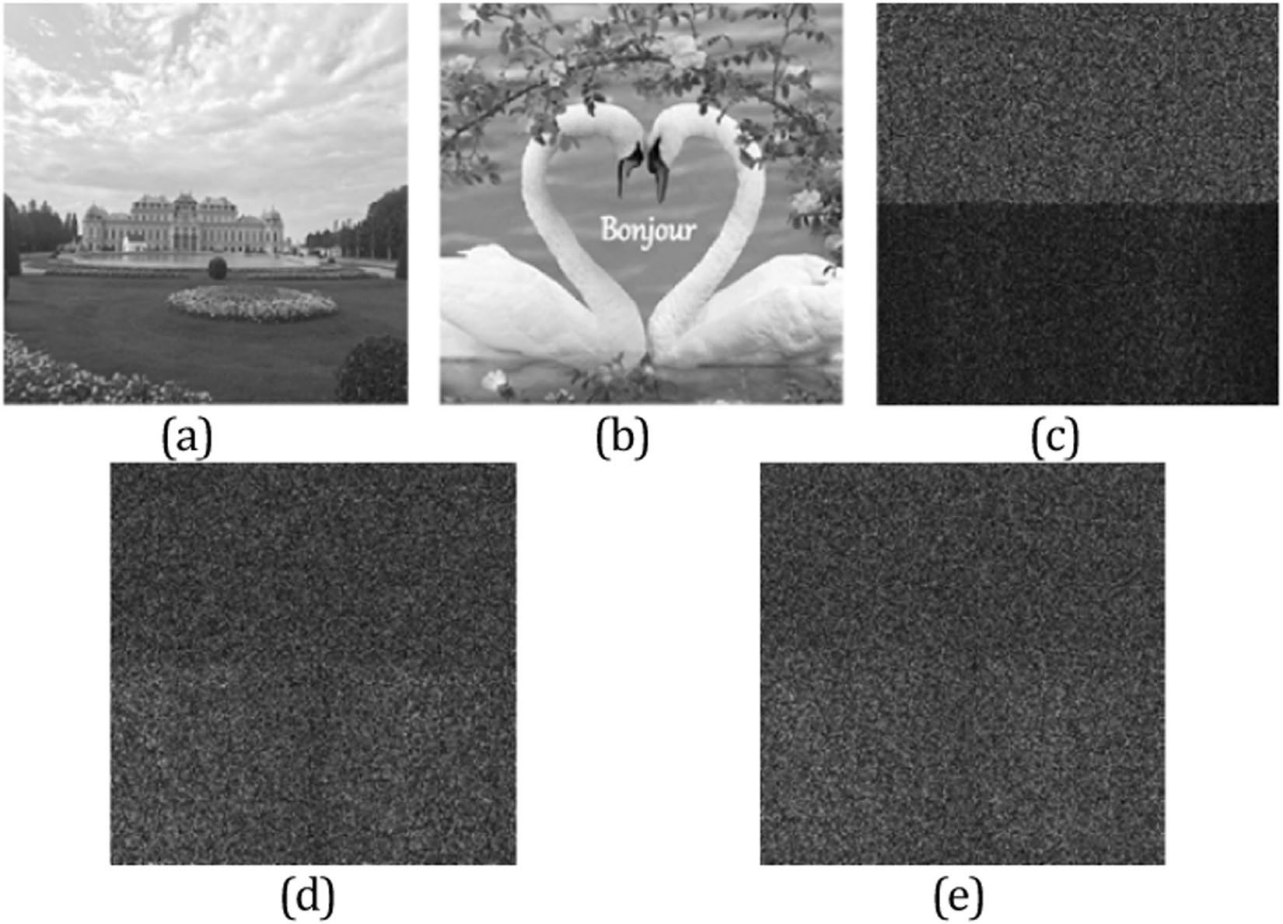
The test of known plaintext attack: (**a**) original image, (**b**) original image, (**c**) the encrypted data of (**a**,**d**) the encrypted data of (**b**,**e**) the result of known plaintext attack.

The known-plaintext attack is also tested by using the phase retrieval algorithm in extend fractional Fourier transform domain. To simplify the calculation, we chose two secret images having 128 × 128 pixels in the attack experiment and the two images are encrypted by the algorithm proposed in this paper, respectively. The secret images and the corresponding encrypted data are shown in Fig. 6(a–d). In simulation, we suppose that the illegal user usurp the first secret image and its encrypted data. Subsequently, the encrypted data of the second image shown in Fig. 6(d) is attacked by the illegal user by using the known-plaintext attack. In the known plaintext attack experiment, the phase retrieval algorithm is implemented for 1000 iterations. Finally, the attack result is displayed in Fig. 6(e), which the detail information of the second secret image cannot be identified entirely and the attack result is almost a random pattern. Obviously, the known plaintext attack is invalid to our proposed encryption scheme.

## Conclusions

In conclusion, a cryptographic system composed by two independent beam paths is proposed. In this encryption scheme, half of the secret data is encoded by a phase modulation in extend fractional Fourier transform and the other half data is modulated by using tilt Fresnel diffraction. The physical structure parameters of the optical system and the phase delay in tilt Fresnel diffraction can be regarded as the keys of the proposed cryptosystem. Some numerical experiments have been performed to demonstrate the validity and robustness of the presented cryptosystem.
