# Author Correction: Dual paths cryptosystem based on tilt Fresnel diffraction using non-spherical mirror and phase modulation in expanded fractional Fourier transform domain

**DOI:** 10.1038/s41598-020-60491-8

**Published:** 2020-02-27

**Authors:** Hang Chen, Zhengjun Liu, Camel Tanougast, Feifei Liu, Walter Blondel

**Affiliations:** 1 School of Electrical Engineering and Automation, Jiangxi University of Science and Technology, Ganzhou, 341000 China; 2 Centre de Recherche en Automatique de Nancy (CRAN-CNRS, UMR 7039), University de Lorraine, Nancy, 54000 France; 3 Department of Automation Measurement and Control, Harbin Institute of Technology, Harbin, 150001 China; 4 LCOMS, University de Lorraine, Nancy, 57070 France

Correction to: *Scientific Reports* 10.1038/s41598-019-50263-4, published online 21 October 2019

In the original version of this Article, Affiliation 4 was incorrectly given as ‘School of Electrical Engineering and Automation, Jiangxi University of Science and Technology, Ganzhou, 341000, China’. The correct affiliation is listed below.

LCOMS, University de Lorraine, Nancy, 57070, France.

In addition, in the original version of this Article, Camel Tanougast, Feifei Liu and Walter Blondel were incorrectly affiliated with affiliation 2, 4 and 1, respectively. The correct affiliations are listed below:

Affiliation 1

School of Electrical Engineering and Automation, Jiangxi University of Science and Technology, Ganzhou, 341000, China.

Hang Chen, Feifei Liu

Affiliation 2

Centre de Recherche en Automatique de Nancy (CRAN-CNRS, UMR 7039), University de Lorraine, Nancy, 54000, France.

Hang Chen, Walter Blondel

Affiliation 3

Department of Automation Measurement and Control, Harbin Institute of Technology, Harbin, 150001, China.

Zhengjun Liu

Affiliation 4

LCOMS, University de Lorraine, Nancy, 57070, France.

Camel Tanougast

This error has now been corrected in the PDF and HTML versions of the Article.

